# Application of the (fr)AGILE scale in the evaluation of multidimensional frailty in elderly inpatients from internal medicine wards: a cross-sectional observational study

**DOI:** 10.3389/fnagi.2023.1276250

**Published:** 2024-01-05

**Authors:** Ying Ma, Dongxin Sui, Shaozhong Yang, Ningning Fang, Zhihao Wang

**Affiliations:** ^1^Department of Geriatric Medicine, Qilu Hospital (Qingdao), Cheeloo College of Medicine, Shandong University, Qingdao, China; ^2^Department of Respiration, The Second Hospital of Shandong University, Jinan, Shandong, China; ^3^Department of Anesthesiology, Qilu Hospital of Shandong University, Jinan, Shandong, China; ^4^Department of Geriatric Medicine, Qilu Hospital of Shandong University, Jinan, Shandong, China

**Keywords:** frailty, (fr)AGILE scale, elderly patients, cross-sectional observational study, hospitalization expenses

## Abstract

**Background:**

With the rapid growth of an aging global population and proportion, the prevalence of frailty is constantly increasing. Therefore, finding a frailty assessment tool suitable for clinical application by physicians has become the primary link in the comprehensive management of frailty in elderly patients. This study used the (fr)AGILE scale to investigate the frailty status of elderly patients from internal medicine wards and identified relevant factors that affect the severity of frailty.

**Method:**

In this study, 408 elderly inpatients in internal medicine departments of Qilu Hospital of Shandong University from May 2021 to August 2022 were enrolled as research subjects, and a cross-sectional observational study was conducted. Researchers evaluated the frailty based on the (fr)AGILE scale score. The general condition, past medical history, physical examination, laboratory examination, nutrition control score, intervention and treatment measures and other elderly patient information was collected. Logistic regression analysis was used to analyze the relevant factors that affect the severity of frailty and hospitalization costs.

**Results:**

According to the (fr)AGILE scale score, the elderly patients were divided into groups to determine whether they were frail and the severity of the frailty. Among them, 164 patients were in the prefrailty stage, which accounted for 40.2%. There were 188 cases of mild frailty that accounted for 46.1%, and 56 cases of moderate to severe frailty that accounted for 13.7%. Decreased grip strength, elevated white blood cell levels, and low sodium and potassium are independent risk factors affecting the severity of frailty. As the severity of frailty increases, the proportion of sodium, potassium, albumin supplementation as well as anti-infection gradually increases.

**Conclusion:**

Frailty is a common elderly syndrome with a high incidence among elderly patients in internal medicine departments. The main manifestations of frailty vary with different severity levels. Inflammation, anemia, and poor nutritional status can lead to an increase in the severity of frailty as well as blood hypercoagulability, myocardial damage, and additional supportive interventions. This ultimately leads to prolonged hospitalization and increased hospitalization costs.

## Introduction

Frailty is conceptually defined as a clinically recognizable state in which the ability of older people to cope with everyday or acute stressors is compromised by an increased vulnerability brought by age-associated declines in physiological reserve and function across multiple organ systems. Compared with nonfragile elderly people, frail elderly people are more prone to three or more chronic diseases, obesity, insomnia, oral problems, increased risk of adverse consequences such as tumbles, fractures and death, prolonged hospitalization, increased readmission rates and medical expenses, and reduced quality of life and functional status (Archibald et al., 2020; Lal et al., 2020; Chi et al., 2021; Middleton et al., 2022). With the rapid growth of an aging global population and proportion, the prevalence of frailty is constantly increasing and is expected to become one of the most severe challenges to global public health by the 22nd century (Yu et al., 2018; Dent et al., 2019).

Previous research has shown that the management of frailty should include multidisciplinary interventions such as regular screening, clinical evaluation, personalized care, physical exercise, nutritional intervention, medication management and social support with the participation of professional physicians, pharmacists, technicians, and nurses (Dent et al., 2019). For hospitalized elderly patients, integrated management based on clinical evaluation and personalized care has potential effects on delaying or improving frailty (Marcucci et al., 2019). Therefore, finding a frailty assessment tool suitable for clinical application by physicians has become a primary link in the integrated management of frailty in elderly patients in internal medicine wards. There is currently no gold standard for evaluating frailty, and the Fried frailty phenotype and frailty index (FI) are the most representative evaluation tools in the definition of frailty operability. The Fried frailty phenotype includes unexplained weight loss, fatigue, decreased grip strength, decreased walking speed, and decreased physical activity and can be diagnosed as frailty if three conditions are met (Dent et al., 2017; Martin and O'Halloran, 2020). The Fried frailty phenotype is easy to operate and suitable for early risk stratification in the population, but the measurement of grip strength and walking speed may be limited by grip devices, space, time, or patient mobility (Fan et al., 2021). FI considers frailty as a collection of symptoms, healthy behavior, clinical signs, diagnosis, and functional limitations and covers physical, social, psychological, and other fields to effectively identify elderly populations with lower levels of frailty (Mitnitski et al., 2001; Martin and O'Halloran, 2020; Fan et al., 2021). However, an FI assessment takes a long time, and first-time patients must undergo a comprehensive elderly assessment before being assessed for frailty. Patients with previous visits also need to have data extracted from routine medical databases, such as physical examinations, diagnosis, and treatment, which limits its clinical application (Liguori et al., 2020; Martin and O'Halloran, 2020; Fan et al., 2021).

The (fr)AGILE scale is a multidimensional frailty assessment tool used for preventive treatment strategies and is constructed from the 10 most predictive items of mortality in the physical, psychological, nutritional, and socioeconomic fields, including feeling that doing anything requires effort, needing help getting up and down stairs, decreased grip strength, temporal oriented deficits, delayed recall deficit, feeling depressed, weight loss over 4.5 kg in the past year, others helping with eating, financial help from family members, and physical help from family members (Liguori et al., 2020). The evaluators do not need to receive professional training, and the evaluation results are not affected by clinical diagnosis. The evaluation only takes approximately 2.5 min and is suitable for elderly populations in communities and hospitals. It has been applied abroad and shows good internal consistency and interrater reliability in evaluating the frailty status of hospitalized elderly people (Liguori et al., 2020; Curcio et al., 2022). This study applied the (fr)AGILE scale to investigate the frailty status of elderly patients in internal medicine departments and identified the relevant factors that affect the severity of frailty. It also explored the impact of frailty severity on hospitalization expenses.

## Method

### Study design and participants

**Figure 1:**
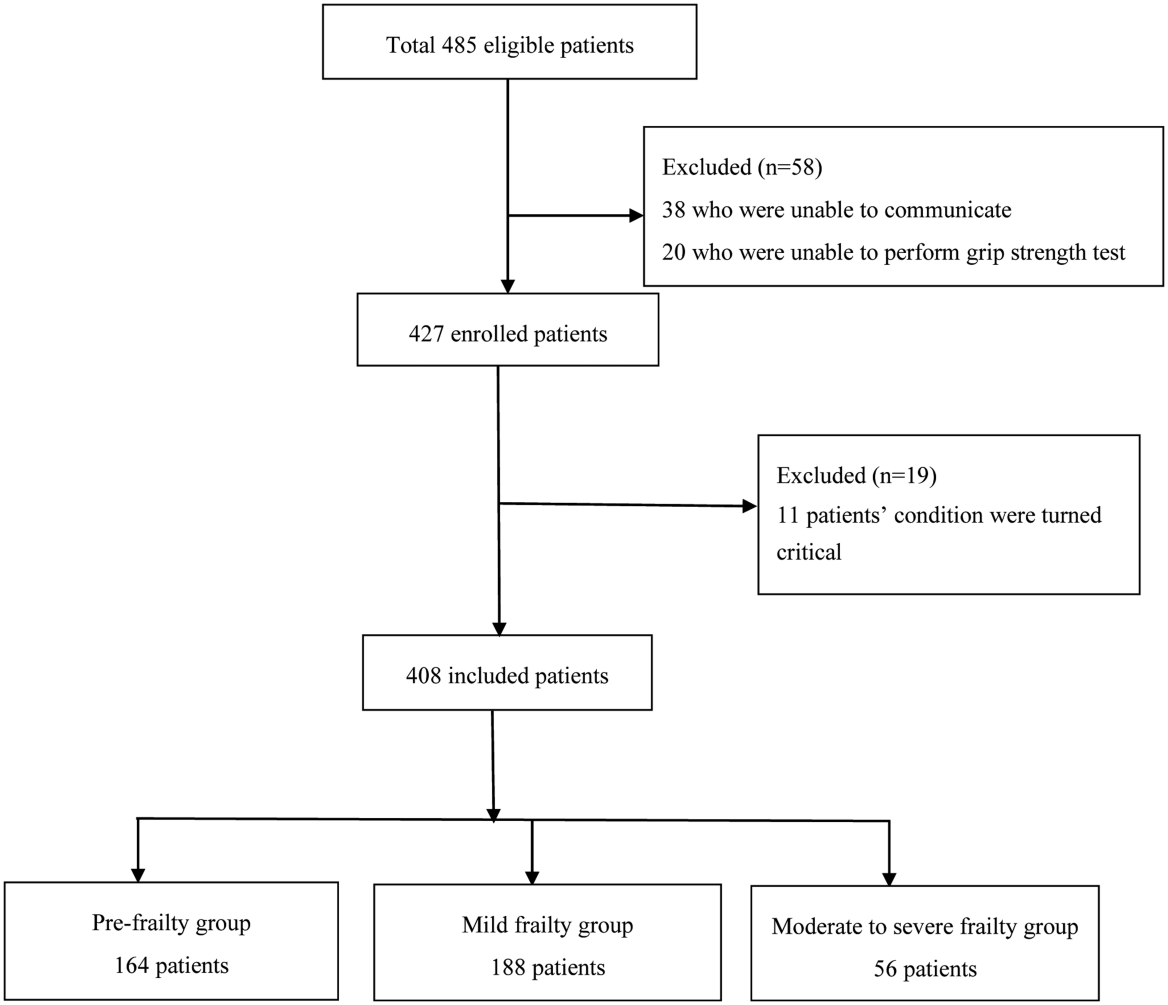
Flow chart of the study population.

In this study, 408 internal medicine inpatients (aged ≥60 years) from Qilu Hospital of Shandong University from May 2021 to August 2022 were enrolled as subjects for a cross-sectional observational study. Among them, there were 217 male patients (53.2%) and 191 female patients (46.8%) with an age range of 60–91 years and an average age of 68.85 ± 5.84 years. All patients signed an informed consent form and voluntarily participated in this study. This study was approved by the Ethics Committee of Qilu Hospital, Shandong University (Ethics Number: 2021-076). Before the evaluation, a detailed explanation of the research purpose, methods, process, and advantages and disadvantages of this study was provided for elderly patients. In addition, this study fully respected the wishes of elderly patients and adhered to the principle of confidentiality for elderly patient information. The inclusion criteria were as follows: internal medicine inpatients aged ≥60 years old who were able to express their feelings and needs and could cooperate with evaluation procedures. The exclusion criteria were as follows: (1) patients who are unable to communicate normally due to cognitive, language, or hearing impairments; (2) patients with severe dysfunction of the brain, heart, lungs, and other organs and in critical condition; (3) patients with limited hand joint activity due to trauma, surgery, rheumatic system diseases, indwelling needles, and other reasons who were unable to measure grip strength. All enrollment and exclusion processes are detailed in Figure 1.

### Data collection

Clinical data were collected through a medical record information query system. The collected information of elderly patients included general conditions, number of children, medicine history, physical examination, laboratory tests, nutrition control status (CONUT) scores, and intervention and treatment measures. The laboratory examinations in this study included complete blood count, coagulation function, serum biochemistry, liver function, renal function, and electrolytes examination, etc.

Researchers scored the (fr)AGILE scale, and the scoring criteria are detailed in Table 1. The (fr)AGILE scale scores 0 for no frailty, 1–2 for prefrailty, 3–4 for mild frailty, and 5–10 for moderate to severe frailty. After evaluation of the scale is completed, the answers of elderly patients are summarized and organized. Two researchers separately recorded the above items, and communicated with those who had objections. If no agreement could be reached then the third researcher decided. The final and total scores of each item were recorded in the elderly patient information table, and the evaluation materials were properly maintained.

**Table 1:** (fr)AGILE scale.

Item	Yes	No	Score
Feel anything is an effort	1	0	
Help up/down stairs	1	0	
Decreased grip strength	1	0	
Temporal oriented deficit	1	0	
Delayed recall deficit	1	0	
Feel depressed	1	0	
Weight loss over 4.5 kg in the last year	1	0	
Help in eating	1	0	
Financial help from family members	0	1	
Physical help from family members	0	1	
Total score			

### Outcomes

The primary outcome of this study was the incidence of frailty in elderly internal medicine patients. The secondary outcomes included hospitalization time and hospitalization expenses.

### Sample size and statistical analysis

The study sample size was calculated using PASS 15 (NCSS, LLC Kaysville, UT, United States). Our small preliminary investigation found that the incidence of frailty was 60%. We assumed that the incidence of frailty was 60% at a two-sided significance level of 0.05 with a confidence interval width of 0.1 and a sample size of 387. To account for 5% loss to follow-up, we recruited at least 408 patients.

SPSS 26.0 was used for statistical analysis of the data. After a data normality test was performed, data conforming to the normal distribution were presented as the mean ± standard deviation ($\bar{\chi }\pm S$). The comparison between two groups was conducted using an independent sample t test, the comparison between multiple groups was conducted using one-way ANOVA, and the pairwise comparison was conducted using the LSD method. Data that did not conform to the normal distribution were all expressed by the median (quartile). The Mann–Whitney U test was used for comparisons between two groups, and the Kruskal-Wallis H test was used for comparisons between multiple groups. The classification data are all represented by the number of cases (%), and intergroup comparisons were made using χ^2^ tests. Logistic regression analysis was used to analyze the relevant factors that affect the severity of frailty and hospitalization costs, and the statistical significance levels were all *p* < 0.05.

## Result

This study included 408 elderly patients in the internal medicine department. According to their (fr)AGILE scale score, elderly patients were divided into groups to determine the level of frailty. Among them, 164 patients were in the prefrailty stage and accounted for 40.2%. There were 188 cases of mild frailty that accounted for 46.1%, and 56 cases of moderate to severe frailty that accounted for 13.7%. Among elderly patients with different degrees of frailty, the proportion of memory impairment was the highest in the prefrailty group and accounted for 50.6%. Among patients with different degrees of frailty, the proportion of those who felt that everything was an effort was highest in the mild frailty group and accounted for 80.3%. The decrease in the proportion of grip strength was the highest among elderly patients in the moderate to severe frailty group and accounted for 96.4% (Table 2).

**Table 2:** Comparison of (fr)AGILE scale evaluation in elderly patients with different degrees of frailty.

Variables	Frailty level			*p*
	Prefrailty group (*n* = 164)	Mild frailty group (*n* = 188)	Moderate to severe frailty group (*n* = 56)	
Feel anything is an effort	76(46.3)	151(80.3)^⁎⁎⁎^	51(91.1)^⁎⁎⁎^	0.000
Help up/down stairs	6(3.7)	32(17.0)^⁎⁎⁎^	43(76.8)^⁎⁎⁎†††^	0.000
Decreased grip strength	72(43.9)	150(79.8)^⁎⁎⁎^	54(96.4)^⁎⁎⁎†^	0.000
Temporal oriented deficit	8(4.9)	27(14.4)^⁎^	30(53.6)^⁎⁎⁎†††^	0.000
Delayed recall deficit	83(50.6)	142(75.5)^⁎⁎⁎^	48(85.7)^⁎⁎⁎^	0.000
Feel depressed	13(7.9)	69(36.7)^⁎⁎⁎^	35(62.5)^⁎⁎⁎††^	0.000
Weight loss over 4.5 kg in the last year	5(3.0)	46(24.5)^⁎⁎⁎^	36(64.3)^⁎⁎⁎†††^	0.000
Help in eating	0(0)	0(0.0)	6(10.7)^⁎⁎††^	0.000
Financial help from family members	0(0)	7(3.7)	2(3.6)	0.035
Physical help from family members	2(1.2)	8(4.3)	0(0)	0.191

Compared with the prefrailty group: ^⁎^*p* < 0.05, ^⁎⁎^*p* < 0.01, ^⁎⁎⁎^*p* < 0.001; Compared with the mild frailty group: ^†^*p* < 0.05, ^††^*p* < 0.01, ^†††^*p* < 0.001.

### Comparison of general conditions of elderly patients with different degrees of frailty

There was no significant difference in age, sex, height, smoking history, drinking history, number of chronic diseases, hypertension, coronary heart disease, or diabetes among the three groups (*p* > 0.05). Compared with the prefrailty group, the mild frailty group showed a significant decrease in grip strength (*p* < 0.001) and a significant increase in hospitalization expenses (*p* < 0.05). Compared with the prefrailty group, the moderate to severe frailty group showed a significant decrease in body weight, BMI, and grip strength (*p* < 0.05 ~ 0.001), while the hospitalization cost and duration increased significantly (*p* < 0.05 ~ 0.01). Compared with the mild frailty group, the grip strength of the moderate to severe frailty group was significantly reduced (*p* < 0.05) (Table 3).

**Table 3:** Comparison of general conditions of elderly patients with different degrees of frailty.

	Frailty level						*p*
	Prefrailty group (*n* = 164)		Mild frailty group (*n* = 188)		Moderate to severe frailty group (N = 56)		
Age (years)	68.16 ± 5.61		69.26 ± 6.03		69.51 ± 5.79		0.194
Gender (male, %)	91(55.5)		94(50.0)		32(57.1)		0.529
Height (m)	1.65 ± 0.07		1.64 ± 0.08		1.62 ± 0.08		0.103
Body weight (kg)	67.66 ± 11.73		64.30 ± 11.96		60.36 ± 10.30^⁎^		0.001
Body mass index (kg/m^2^)	24.71 ± 3.43		23.93 ± 3.86		23.16 ± 4.03^⁎^		0.041
Grip strength (kg)	24.08 ± 7.31		19.09 ± 7.90^⁎⁎⁎^		15.88 ± 8.27^⁎⁎⁎†^		0.000
Smoking history	51(31.1)		47(29.8)		25(44.6)		0.149
Drinking history	48(34.8)		94(50.0)		20(35.7)		0.218
Number of children	2.00(1.00, 2.00)		2.00(1.00, 3.00)		2.00(1.00, 3.00)		0.143
Number of chronic diseases	2.00(2.00, 3.00)		3.00(2.00, 4.00)		2.00(1.00, 3.00)		0.513
History of hypertension	91(55.5)		90(47.9)		23(41.1)		0.166
History of coronary heart disease	66(40.2)		80(42.6)		17(30.4)		0.298
History of diabetes	65(39.6)		62(33.0)		19(33.9)		0.493
Hospitalization expenses (USD)	1871.4(1185.7, 3171.4)		2242.9(1485.7, 3671.4)^*^		2757.1(1742.9, 4985.7)^**^		0.003
Hospitalization time (days)	8.00(6.00, 12.00)		9.00(7.00, 12.00)		10.00(7.00, 14.00)^*^		0.019

Compared with the prefrailty group: ^*^p < 0.05, ^**^p < 0.01, ^***^p < 0.001; compared with the mild frailty group: ^†^*p* < 0.05, ^††^*p* < 0.01, ^†††^*p* < 0.001.

### Comparison of laboratory examinations in elderly patients with different degrees of frailty

There was no statistically significant difference in platelet count (PLT), alanine aminotransferase (ALT), aspartate aminotransferase (AST), homocysteine (Hcy), blood glucose (GLU), blood urea nitrogen (BUN), blood creatinine (Cr), total cholesterol (TC), high-density lipoprotein cholesterol (HDL-C), low-density lipoprotein cholesterol (LDL-C), triglycerides (TG), lactate dehydrogenase (LDH), potassium ions (K^+^), magnesium ions (Mg^2+^) among the prefrailty group, mild frailty group, and moderate to severe frailty group (*p* > 0.05). Compared with the prefrailty group, the mild frailty group showed a significant decrease in hemoglobin and serum albumin (ALB) (*p* < 0.05–0.01) and a significant increase in D-dimer (*p* < 0.01). Compared with the prefrailty group, the moderate to severe frailty group showed a significant decrease in lymphocyte (LYMT), red blood cell (RBC), hemoglobin, ALB, creatine kinase (CK), sodium ions (Na^+^), and calcium ions (Ca^2+^) (*p* < 0.05 ~ 0.001) and a significant increase in D-dimer, cardiac troponin I (cTnI), ischemic modified albumin (IMA), and CONUT scores (*p* < 0.01 ~ 0.001). Compared with the mild frailty group, the moderate to severe frailty group had a significantly decrease in ALB and Na^+^ levels (*p* < 0.05–0.01), while white blood cell (WBC), neutrophil count (NEUT), cTnI, and IMA levels were significantly increased (*p* < 0.05–0.01) (Table 4).

**Table 4:** Comparison of laboratory examinations in elderly patients with different degrees of frailty.

	Frailty level			*p*
	Prefrailty group (*n* = 164)	Mild frailty group (*n* = 188)	Moderate to severe frailty group (*n* = 56)	
WBC(×10^9^/L)	5.98(4.84, 7.26)	5.68(4.53, 7.15)	6.81(5.07, 9.02)^††^	0.011
NEUT(×10^9^/L)	3.86(2.78, 4.94)	3.59(2.78, 4.76)	4.62(2.99, 7.09)^†^	0.020
LYMT(×10^9^/L)	1.52(1.18, 1.89)	1.39(1.05, 1.82)	1.28(0.83, 1.91)^*^	0.037
RBC (×10^12^/L)	4.36(3.99, 4.68)	4.21(3.78, 4.61)	4.00(3.47, 4.38)^***^	0.000
Hb(g/L)	133.00 (120.00, 143.00)	127.00 (111.75, 138.25)^*^	118.00 (105.00, 133.00)^***^	0.000
PLT(×10^9^/L)	213.00 (168.50, 249.75)	217.00 (175.75, 279.25)	222.00 (181.00, 308.00)	0.326
D-dimer(μg/mL)	0.26(0.12, 0.66)	0.53(0.27, 1.07)^**^	0.83(0.37, 1.38)^***^	0.000
ALT(U/L)	15.00(11.00, 19.50)	14.00(10.00, 25.00)	17.00(10.00, 37.00)	0.588
AST(U/L)	17.00(15.00, 22.00)	19.00(15.00, 24.50)	17.50(13.75, 36.50)	0.362
ALB(g/L)	42.00 (38.63, 44.58)	40.35 (36.03, 43.58)^**^	37.80 (32.55, 41.05)^***†^	0.000
Hcy(μmol/L)	11.60(9.95, 14.60)	11.90(9.55, 14.20)	13.70(9.90, 19.35)	0.101
GLU(mmol/L)	5.11(4.58, 6.75)	5.30(4.69, 6.65)	5.55(4.48, 7.20)	0.749
BUN(mmol/L)	5.10(4.23, 6.20)	5.40(4.30, 6.80)	5.75(4.80, 7.88)	0.061
Cr(μmol/L)	68.00(59.00, 81.00)	67.50(56.25, 77.75)	63.50(53.75, 89.00)	0.769
TC(mmol/L)	4.09(3.17, 4.90)	4.19(3.49, 4.90)	4.25(3.19, 5.15)	0.604
HDL-C(mmol/L)	1.15(0.95, 1.36)	1.14(0.97, 1.32)	1.08(0.76, 1.32)	0.210
LDL-C(mmol/L)	2.22(1.72, 2.93)	2.47(1.99, 2.94)	2.52(1.74, 3.03)	0.373
TG(mmol/L)	1.21(0.86, 1.63)	1.14(0.84, 1.57)	1.22(0.88, 1.80)	0.522
CK(U/L)	65.00(47.50, 110.50)	59.00(38.00, 90.00)	37.50(24.00, 80.25)^*^	0.014
cTnI(ng/L)	3.98(2.39, 6.28)	4.35(2.71, 7.52)	7.29(3.65, 12.32)^***†^	0.001
LDH(U/L)	206.50 (180.25, 237.00)	205.50 (188.00, 245.50)	194.50 (171.75, 256.75)	0.442
IMA(U/mL)	74.80 (69.60, 81.40)	77.45 (71.03, 86.48)	83.90 (78.18, 90.58)^***††^	0.000
K^+^(mmol/L)	4.10 ± 0.36	4.04 ± 0.42	4.01 ± 0.50	0.308
Na^+^(mmol/L)	141.00 (139.00, 143.00)	141.00 (138.00, 142.00)	138.00 (133.00, 142.00)^***††^	0.001
Ca^2+^(mmol/L)	2.27(2.17, 2.35)	2.23(2.15, 2.31)	2.21(2.11, 2.29)^*^	0.012
Mg^2+^(mmol/L)	0.90(0.84, 0.95)	0.88(0.83, 0.93)	0.90(0.82, 0.96)	0.262
CONUT score	2.00(1.00, 3.00)	2.00(1.00, 4.00)	3.00(1.00, 5.75)^**^	0.006

Compared with the prefrailty group: ^*^p < 0.05, ^**^p < 0.01, ^***^p < 0.001; compared with the mild frailty group: ^†^*p* < 0.05, ^††^*p* < 0.01, ^†††^*p* < 0.001. WBC, white blood cell; NEUT, neutrophil count; LYMT, lymphocyte count; RBC, red blood cell; Hb, hemoglobin; PLT, platelet count; ALT, alanine aminotransferase; AST, aspartate aminotransferase; ALB, serum albumin; Hcy, homocysteine; GLU, blood glucose; BUN, blood urea nitrogen; Cr, blood creatinine; TC, total cholesterol; HDL-C, high-density lipoprotein cholesterol; LDL-C, low-density lipoprotein cholesterol; TG, triglycerides; CK, creatine kinase; cTnI, cardiac troponin I; LDH, lactate dehydrogenase; IMA, ischemic modified albumin; K+, potassium ions; Na+, sodium ions; Ca2+, calcium ions; Mg2+, magnesium ions; CONUT, nutritional control status.

### Comparison of supports and treatments among elderly patients with different degrees of frailty

There was no statistically significant difference in calcium and magnesium supplementation or blood transfusion among the prefrailty group, mild frailty group, and moderate to severe frailty group (*p* > 0.05). Compared with the prefrailty group, the proportion of anti-infection in the mild frailty group significantly increased (*p* < 0.05). Compared with the prefrailty group, the proportion of sodium, potassium, and albumin supplementation significantly increased in the moderate to severe frailty group (*p* < 0.05 ~ 0.001). Compared with the mild frailty group, the proportion of albumin supplementation in the moderate to severe frailty group was significantly increased (*p* < 0.001) (Table 5).

**Table 5:** Comparison of treatments among elderly patients with different degrees of frailt.

	Frailty level			*p*
	Prefrailty group (*n* = 164)	Mild frailty group (*n* = 188)	Moderate to severe frailty group (*n* = 56)	
Sodium supplementation	40(24.4)	50(26.6)	24(42.9)^*^	0.046
Potassium supplementation	60(36.6)	89(47.3)	37(66.1)^**^	0.002
Calcium supplementation	59(36.0)	82(43.6)	24(42.9)	0.365
Magnesium supplementation	36(22.0)	49(26.1)	17(30.4)	0.508
Serum albumin supplementation	11(6.7)	15(8.0)	18(32.1)^***†††^	0.000
Blood transfusion	4(2.4)	10(5.3)	6(10.7)	0.058
Anti-infection	57(34.8)	90(47.9)^*^	29(51.8)	0.040

Compared with the prefrailty group: ^*^p < 0.05, ^**^p < 0.01, ^***^p < 0.001; compared with the mild frailty group: ^†^*p* < 0.05, ^††^*p* < 0.01, ^†††^*p* < 0.001.

### Logistic regression analysis of clinical associated risk factors for frailty

A univariate unordered logistic regression model was constructed using laboratory examination results, clinical features, and supportive interventions as covariates and frailty as the dependent variable. Results showed that the effects of body weight, body mass index (BMI), grip strength, potassium supplementation, blood transfusion, anti-infection, WBC, RBC, hemoglobin, ALT, HDL-C, Hcy, BUN, Na^+^, and CONUT scores on frailty were statistically significant in the model (*p* < 0.05). The results of multivariate unordered logistic regression model showed that grip strength (OR: 0.885, 95% CI: 0.758–1.044, *p* = 0.000) was negatively correlated with mild frailty, and grip strength (OR: 0.855, 95% CI: 0.791–0.923, *p* = 0.000) Na^+^ (OR: 0.863, 95% CI: 0.746–0.998, *p* = 0.047) was negatively correlated with moderate to severe frailty, while potassium supplementation (OR: 2.795, 95% CI: 1.054–7.408, *p* = 0.039) and WBC count (OR: 1.305, 95% CI: 1105–1.543, *p* = 0.002) were positively correlated with moderate to severe frailty (Tables 6, 7).

**Table 6:** Logistic regression analysis of clinical associated risk factors for frailty (prefrailty *vs* mild frailty).

	Univariate			Multivariate		
	*β*	OR(95%CI)	*P*	*β*	OR(95%CI)	*P*
Weight	−0.024	0.976(0.956, 0.996)	0.018			
BMI	−0.056	0.945(0.887, 1.007)	0.080			
Grip strength	−0.084	0.920(0.890, 0.950)	0.000	−0.122	0.885(0.758, 1.044)	0.000
Potassium supplementation	0.453	1.572(0.985, 2.509)	0.058			
Blood transfusion	0.868	2.382(0.619, 9.164)	0.207			
Anti infection	0.541	1.718(1.074, 2.749)	0.024			
WBC	−0.001	0.999(0.905, 1.102)	0.981			
RBC	0.009	1.009(0.980, 1.038)	0.562			
Hb	−0.012	0.988(0.977, 0.999)	0.026			
ALT	0.008	1.008(1.000, 1.017)	0.063			
HDL-C	0.059	1.060(0.838, 1.343)	0.626			
Hcy	−0.008	0.992(0.947, 1.039)	0.726			
BUN	0.077	1.080(0.981, 1.189)	0.117			
Na^+^	−0.026	0.974(0.907, 1.046)	0.468			
CONUT score	0.123	1.131(0.990, 1.292)	0.070			

BMI, body mass index; WBC, white blood cell; RBC, red blood cell; Hb, hemoglobin; ALT, alanine aminotransferase; HDL-C, high-density lipoprotein cholesterol; Hcy, homocysteine; BUN, blood urea nitrogen; Na^+^, sodium ion; CONUT, nutrition control status; OR, odds ratio; 95% CI, 95% confidence interval.

**Table 7:** Logistic regression analysis of clinical associated risk factors for frailty (prefrailty *vs* moderate to severe frailty).

	Univariate			Multivariate		
	*β*	OR(95%CI)	*p*	*β*	OR(95%CI)	*p*
Weight	−0.056	0.946(0.915, 0.977)	0.001			
Grip strength	−0.139	0.870(0.829, 0.913)	0.000	−0.157	0.855(0.791, 0.923)	0.000
BMI	−0.117	0.890(0.804, 0.984)	0.023			
Potassium supplementation	1.215	3.371(1.680, 6.765)	0.001	1.028	2.795(1.054, 7.408)	0.039
Blood transfusion	1.671	5.317(1.219, 23.190)	0.026			
Anti-infection	0.660	1.935(0.989, 3.785)	0.054			
WBC	0.174	1.190(1.064, 1.332)	0.002	0.267	1.305(1.105, 1.543)	0.002
RBC	−0.727	0.484(0.313, 0.746)	0.001			
Hb	−0.025	0.975(0.961, 0.989)	0.001			
ALT	0.010	1.010(1.000, 1.019)	0.046			
HDL-C	−1.090	0.336(0.118, 0.961)	0.042			
Hcy	0.060	1.062(1.011, 1.116)	0.016			
BUN	0.144	1.155(1.038, 1.285)	0.008			
Na^+^	−0.206	0.814(0.742, 0.893)	0.000	−0.147	0.863(0.746, 0.998)	0.047
CONUT score	0.339	1.404(1.189, 1.659)	0.000			

Abbreviations were shown in Table 6.

### Logistic regression analysis of clinical associated risk factors for hospitalization expenses

The univariate logistic regression analysis showed that WBC, NEUT, AST, ALB, TC, LDL-C, Hcy, LDH, IMA, number of children, (fr)AGILE score, Na^+^, potassium, calcium, magnesium, albumin, blood transfusion, anti-infection, and diabetes were statistically significant in the model (*p* < 0.05). Further multivariate logistic regression analysis showed that ALB (OR: 0.965, 95% CI: 0.952–0.978, *p* = 0.000), sodium supplementation (OR: 3.230, 95% CI: 1.318–7.915, *p* = 0.010), potassium supplementation (OR: 3.443, 95% CI: 1.643–7.211, *p* = 0.001), and anti-infection (OR: 3.076, 95% CI: 1.694–5.586, *p* = 0.000) were all influencing factors for hospitalization expenses (Table 8).

**Table 8:** Logistic regression analysis of clinical associated risk factors for hospitalization expenses.

	Univariate			Multivariate		
	*β*	OR(95%CI)	*P*	*β*	OR(95%CI)	*P*
WBC	0.101	1.107(1.015, 1.207)	0.022			
NEUT	0.132	1.141(1.032, 1.261)	0.010			
AST	0.011	1.012(1.001, 1.023)	0.038			
ALB	−0.069	0.934(0.893, 0.976)	0.002	−0.036	0.965(0.952, 0.978)	0.000
TC	0.221	1.247(1.016, 1.529)	0.034			
LDL-C	0.318	1.374(1.052, 1.796)	0.020			
Hcy	0.050	1.051(1.007, 1.097)	0.022			
LDH	0.004	1.004(1.000, 1.007)	0.048			
IMA	0.029	1.030(1.004, 1.056)	0.024			
Number of children	0.363	1.437(1.163, 1.776)	0.001			
AGILE score	0.180	1.197(1.023, 1.400)	0.025			
Sodium supplementation	1.191	3.290(1.979, 5.470)	0.000	1.173	3.230(1.318, 7.915)	0.010
Potassium supplementation	1.216	3.372(2.159, 6.267)	0.000	1.236	3.443(1.643, 7.211)	0.001
Calcium supplementation	0.538	1.713(1.107, 2.650)	0.016			
Magnesium supplementation	0.937	2.554(1.526, 4.273)	0.000			
Serum albumin supplementation	1.845	6.331(2.572, 15.582)	0.000			
Blood transfusion	1.528	4.608(1.289, 16.473)	0.019			
Anti-infection	0.922	2.515(1.620, 3.904)	0.000	1.124	3.076(1.694, 5.586)	0.000
Diabetes	−0.777	0.460(0.292, 0.724)	0.001			

NEUT, neutrophil count; AST, aspartate aminotransferase; ALB, serum albumin; TC, total cholesterol; LDL-C, low-density lipoprotein cholesterol; LDH, lactate dehydrogenase; IMA, ischemic modified albumins; other abbreviations were shown in Table 6.

## Discussion

In this study, the (fr)AGILE scale score was used to conduct a cross-sectional observational study on the frailty of elderly patients in the internal medicine department. Frailty was very common among elderly patients with approximately 6 out of every 10 patients having frailty. The (fr)AGILE scale, as a fast and reliable multidimensional frailty assessment tool, can help physicians quickly identify the frailty status of elderly patients.

The internal medicine ward is the primary location for the clinical diagnosis and treatment of elderly patients with chronic diseases. Almeida et al. (2019) used the Tilburg frailty index to evaluate elderly patients admitted to the internal medicine department, and results showed that the frailty prevalence rate of elderly patients was 58.5%. In this study, we used the (fr)AGILE scale to evaluate the frailty of 408 elderly patients aged 60 and above in internal medicine departments (including the Department of Gastroenterology, Endocrinology, Respiratory, Cardiology, Rheumatology, and Hepatology). Results showed that the prevalence of prefrailty and frailty in elderly patients was 40.2% and 59.8%, respectively. Forti et al. (2014) used the Osteoporosis Fracture Research Index to evaluate 470 elderly patients in an internal medicine ward. Results showed that the prevalence rates of prefrailty and frailty in elderly patients were 30 and 50%, respectively. Our results are consistent with those mentioned above but slightly higher. This is because there are differences in the selection of research subjects and frailty assessment tools among different studies. In addition, the above studies indicate that for the same elderly population, the prevalence of frailty using multidimensional frailty assessment tools is significantly higher than that using single-dimensional frailty assessment tools (Liang et al., 2019).

A large body of research has revealed that frailty is related to cognitive function in later life and that cognitive function and frailty interact in an age-related decline cycle. The frailty and cognitive impairment may share similar etiologies. For example, progression of frailty is associated with incident Alzheimer’s disease (AD) and an accelerated rate of cognitive decline in the elderly (Boyle et al., 2010). Over 20 neuro-inflammatory markers have been reported to possibly have an association with both physical frailty and a decrease in cognitive functions (Sargent et al., 2018). A longitudinal study in China demonstrated that older adults with subjective cognitive decline were more likely to have pre-frailty or frailty (Hsieh et al., 2018). The frail elderly people are more likely to have cognitive decline and memory decline than robust ones (Nishiguchi et al., 2015). Previous study found that the prevalence of cognitive impairment among the pre-frailty was 47.41% (Sharifi et al., 2021), which was consistent with our result. Cognitive impairment might be related to frailty phenotype and the probability of cognitive impairment in pre-frail subjects was higher than normo-cognitive older adults. This indicates that cognitive screening for elderly and frail individuals is of great significance, and we should pay attention to cognitive decline.

Due to a series of factors, such as the intensification of population aging and the surge in demand for medical treatment among people, the total and *per capita* health expenses in China are on the rise and the problem of poverty caused by illness and a return to poverty due to illness is very prominent. Previous research determined that frailty can lead to a significant increase in hospitalization costs. Rubens et al. (2022) conducted a retrospective study on cancer patients hospitalized in the United States from 2005 to 2014 and found that compared to nonfragile patients, frail patients had significantly higher hospital stays, mortality rates, and hospitalization costs. Wong et al. (2023) and Kwak et al. (2022) found a significant increase in hospitalization costs for frail elderly patients with hip fractures. Sy et al. (2020) found that the total medical expenses of hemodialysis patients with frailty increased by 22% compared to nonfrail patients with outpatient and inpatient expenses increasing by 24.6% and 62.9%, respectively. Rosiello et al. (2022) found in their study of nonmetastatic renal cell carcinoma patients undergoing partial nephrectomy that frail patients have longer hospital stays and higher hospitalization costs than nonfrail patients. Our research indicated a positive correlation between frailty and hospitalization costs. As the degree of frailty increases, the hospitalization time and hospitalization costs of elderly patients are significantly prolonged. To clarify the reasons for the increase in hospitalization costs for frail elderly patients, we conducted a logistic regression analysis. The results showed that the increase in hospitalization costs for elderly patients was related to sodium, potassium, calcium, magnesium, and albumin supplementation as well as anti-infection, blood transfusion, weekend hospitalization, needing help getting up and down stairs, and weight loss of over 4.5 kg in the past year. In addition, elevated albumin levels can reduce hospitalization costs.

Recently, malnutrition has been identified as an important influencing factor for frailty in elderly patients with 33.5% of hospitalized elderly patients experiencing both frailty and malnutrition (Ni Lochlainn et al., 2021; Sharma et al., 2021). This study found that elderly people with poor nutritional status had a higher degree of frailty. As the degree of frailty increases, the levels of BMI, RBC, Hb, ALB, CK, Na^+^, and Ca^2+^ in elderly patients significantly decrease while the proportion of sodium, potassium, and albumin supplementation significantly increases. The degree of frailty is positively correlated with the CONUT score. In the logistic regression analysis of frailty, the need for potassium supplementation and a decrease in Na^+^ levels were independent risk factors for frailty. This is consistent with previous studies. Mailliez et al. (2020) found that Hb and ALB levels in frail populations were relatively low. Xu et al. (2020) found that elderly people with a lower BMI had a higher risk of frailty. Fujisawa et al. (2022) found that elderly people with higher levels of frailty were more prone to developing hyponatremia, hypokalemia, and hypocalcemia. Asaoka et al. (2020) found that frail elderly people have higher CONUT scores. Yaku et al. (2020) found that patients with hypoalbuminemia and hyponatremia had a 76% and 49% increased risk of frailty, respectively.

The above results indicate that the increasing demand for supportive treatment, malnutrition, and decreased ability to engage in daily activities in frail elderly patients are the main reasons for the increase in hospitalization costs. Therefore, physicians should promptly identify the frailty status of elderly patients and develop comprehensive management plans, including nutritional support, personalized care, medication management, and physical exercise, to improve the quality of life of elderly patients, reduce hospitalization costs, reduce personal and family medical expenses, and reduce the social medical burden.

## Conclusion

Frailty is a common elderly syndrome with a high incidence among elderly patients in internal medicine wards. The main manifestations of frailty vary with different severity levels. Inflammation, anemia, and poor nutritional status are associated with severity of frailty, affecting blood hypercoagulability, myocardial damage, and additional supportive interventions, as well as prolonged hospitalization and increased hospitalization costs. Decreased grip strength, elevated white blood cell levels, and low sodium and potassium are independent risk factors affecting the severity of frailty. As the severity of frailty increases, the proportion of sodium, potassium and albumin supplementation as well as anti-infection gradually increases. In addition, elevated serum albumin levels is related to decreased hospitalization costs.

## Data availability statement

The original contributions presented in the study are included in the article/supplementary material, further inquiries can be directed to the corresponding author.

## Ethics statement

The studies involving humans were approved by this study was approved by the Ethics Committee of Qilu Hospital, Shandong University (Ethics Number: 2021-076). The studies were conducted in accordance with the local legislation and institutional requirements. The participants provided their written informed consent to participate in this study. Written informed consent was obtained from the individual(s) for the publication of any potentially identifiable images or data included in this article.

## Author contributions

YM: Data curation, Formal analysis, Methodology, Writing – original draft. DS: Investigation, Methodology, Software, Writing – review & editing. SY: Investigation, Methodology, Project administration, Software, Writing – original draft. NF: Data curation, Investigation, Writing – original draft. ZW: Formal analysis, Funding acquisition, Project administration, Resources, Writing – original draft.
